# Multidisciplinary Single Stage Surgical Management of Advanced Breast Carcinoma With Concomitant Left Atrial Myxoma

**DOI:** 10.1093/icvts/ivag252

**Published:** 2026-09-21

**Authors:** Shriya Haval, Anurag Garg, Ranjit Pawar, Manoj Dongare

**Affiliations:** Department of Cardiovascular and Thoracic Surgery, Dr D Y Patil Vidyapeeth, Pune, Maharashtra 411018, India; Department of Cardiovascular and Thoracic Surgery, Dr D Y Patil Vidyapeeth, Pune, Maharashtra 411018, India; Department of Cardiovascular and Thoracic Surgery, Dr D Y Patil Vidyapeeth, Pune, Maharashtra 411018, India; Department of Surgical Oncology, Dr D Y Patil Vidyapeeth, Pune, Maharashtra 411018, India

**Keywords:** left atrial myxoma, breast carcinoma, multidisciplinary, interatrial septal reconstruction

## Abstract

Primary cardiac tumours are rare, with atrial myxomas accounting for approximately 50% of benign cardiac tumours. In patients with malignancy, intracardiac masses pose a diagnostic challenge because distinguishing primary tumours from metastases directly influences management. A 58-year-old woman with locally advanced invasive mucinous breast carcinoma developed a progressive fungating breast mass despite neoadjuvant chemotherapy and radiotherapy. Routine echocardiography incidentally revealed a large left atrial mass attached to the interatrial septum. Cardiac MRI and FDG PET-CT suggested a cardiac neoplasm but could not exclude metastasis. Given the embolic risk and urgent need for oncologic surgery, a multidisciplinary team performed single-stage toilet mastectomy, atrial mass excision with interatrial septal reconstruction and split-thickness skin grafting under one anaesthetic. Histopathology confirmed glandular atrial myxoma. Single-stage multidisciplinary surgery may safely reduce treatment delay, embolic risk, repeated anaesthesia, and hospitalization in carefully selected patients.

## INTRODUCTION

Primary cardiac tumours are rare, with atrial myxomas accounting for nearly half of benign tumours.^1^ In patients with malignancy, differentiating myxoma from cardiac metastasis is essential, as metastatic cardiac involvement is more common than primary cardiac tumours. Although breast carcinoma commonly metastasizes to the bone, lung, liver, and brain, cardiac involvement is uncommon.^2^ The coexistence of breast carcinoma and left atrial myxoma is extremely rare and may pose significant diagnostic and therapeutic challenges, with a single-stage multidisciplinary approach may help reduce treatment delay, embolic risk, and repeated anaesthesia exposure.

## CASE PRESENTATION

A 58-year-old female with type 2 diabetes mellitus presented with a progressively enlarging fungating right breast mass. She had previously been diagnosed with invasive mucinous adenocarcinoma and treated with chemotherapy and radiotherapy; however, the lesion progressed, requiring palliative toilet mastectomy. Preoperative echocardiography incidentally revealed a large left atrial mass (53 × 32 mm) attached to the interatrial septum, suggestive of myxoma. Cardiac MRI and PET-CT confirmed an FDG-avid intracardiac lesion, although cardiac metastasis could not be excluded. The preoperative course was complicated by poor glycaemic control, urinary tract infection, and anaemia requiring optimization. Given the embolic risk and need for oncologic surgery, a multidisciplinary team planned a single-stage combined procedure.

### Operative procedure

All procedures were performed sequentially under the same general anaesthesia.

**Oncologic procedure (Figure 1A):** Toilet mastectomy with axillary lymph node dissection was performed for the fungating right breast carcinoma. The chest wall wound was temporarily covered with a sterile dressing, and the patient was repositioned for cardiac surgery.

**Figure 1.**
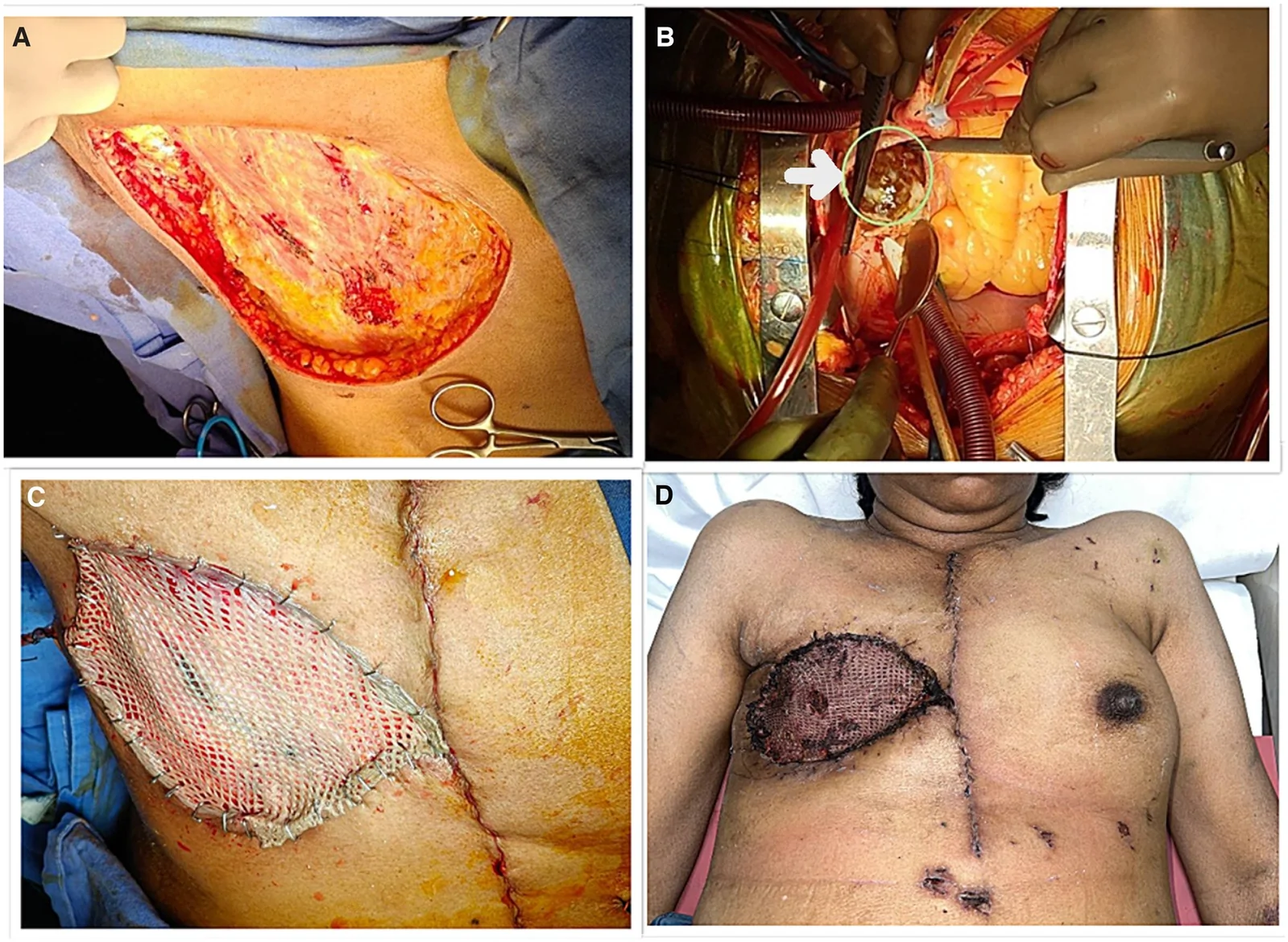
Clinical and Intraoperative Images. (A) Chest wall defect after right toilet mastectomy. (B) Left atrial mass (arrow) attached to the interatrial septum. (C) Chest wall reconstruction with STSG. (D) Postoperative day 10: healed sternal wound and successful skin graft uptake.

**Cardiac procedure (Figure 1B):** Median sternotomy and cardiopulmonary bypass were established using standard aortic and bicaval cannulation. Following cross-clamping and cardioplegic arrest, left atriotomy revealed a large mass eroding the interatrial septum. En bloc excision with Dacron patch reconstruction was performed. Cardiopulmonary bypass and cross-clamp times were 128 and 83 min, respectively. Frozen section favoured metastasis. The patient was weaned uneventfully, and sternal closure was performed with sternal bands due to the post-radiotherapy osteoporotic sternum.

**Plastic surgery (Figure 1C):** Flap reconstruction was deferred because of possible metastasis and future radiotherapy. The mastectomy defect was covered with a split-thickness skin graft [STSG] from the right thigh.

### Postoperative course

The patient remained haemodynamically stable postoperatively. Oral acenocoumarol was initiated on postoperative day 2 after adequate haemostasis. Therapeutic heparin bridging was avoided because of persistent oozing from the skin-graft donor site and concern for wound-related bleeding. Vitamin K antagonist therapy was preferred following extensive atrial septal reconstruction, allowing INR-guided dose adjustment during the early postoperative period. Recovery was uneventful, with satisfactory graft uptake, stable sternal wound healing, and no thromboembolic complications **Figure 1D**.

Frozen section favoured metastatic adenocarcinoma; however, glandular atrial myxoma contains epithelial-lined glandular structures within myxoid stroma and can closely mimic metastatic adenocarcinoma. Definitive paraffin sections demonstrated characteristic myxoid stroma with benign stellate cells and glandular differentiation, confirming glandular atrial myxoma and excluding metastatic carcinoma (**Table 1**). This highlights the limitations of frozen-section diagnosis in rare histological variants and the importance of definitive histopathological evaluation.

**Table 1.** Histopathology Report

Specimen	Finding	
**Cardiac mass**	Left atrial myxoma—glandular variant	
**Breast tumour type**	High-grade mucinous carcinoma with goblet cell-like and apocrine differentiation	
	**Tumour size**	9.5 × 8 × 3.8 cm
	**Skin involvement**	Present with ulceration
	**Lymphovascular invasion**	Present
	**Lymph node**	2/14 positive
	**Extranodal extension**	Present
	**Residual Cancer Burden**	RCB III
	**Pathological stage**	ypT4 ypN1a Mx

## DISCUSSION

In patients with malignancy, intracardiac masses pose a diagnostic challenge because cardiac metastases are more common than primary cardiac tumours. Although echocardiography and cardiac MRI aid diagnosis, definitive differentiation between myxoma and metastasis may require histopathological confirmation. In our patient, the decision for single-stage surgery was made after multidisciplinary discussion involving cardiac surgery, surgical oncology, anaesthesia, and plastic surgery. Factors favouring a combined procedure included the large, mobile left atrial mass attached to the interatrial septum with significant embolic potential, rapidly progressive fungating breast carcinoma despite neoadjuvant treatment, preserved cardiopulmonary reserve, and the desire to avoid repeated general anaesthesia. A staged approach could have exposed the patient to systemic embolization during breast surgery or further oncological progression while awaiting cardiac surgery.

Reported cases of cardiac tumours associated with malignancy are limited. Meir et al^3^ described breast carcinoma with left atrial myxoma managed by staged surgery, while Miyamoto et al^4^ reported metastatic renal cell carcinoma presenting as a left atrial mass mimicking myxoma and treated with staged excision. In contrast, our patient underwent successful single-stage combined surgery for advanced breast carcinoma and a large left atrial mass, highlighting the rarity of this approach. Although combined surgery increases operative duration and perioperative complexity, the anticipated benefits—including avoidance of repeated hospitalization and anaesthesia, reduction of embolic risk, earlier oncologic control, and definitive treatment during a single admission—were considered to outweigh the additional operative burden.

## CONCLUSION

This case shows successful single-stage multidisciplinary management of advanced fungating breast carcinoma with a concomitant left atrial mass. Careful patient selection remains fundamental to successful single-stage surgery. In appropriately selected patients with acceptable physiological reserve and coordinated multidisciplinary planning, combined cardiac and oncologic procedures may safely reduce embolic risk, minimise treatment delay, avoid repeated anaesthesia, and provide definitive treatment during a single hospital admission.

## Data Availability

All relevant data supporting the findings of this study are included within the article.
